# Catheter Based Simultaneous Mapping of Cardiac Activation and Motion: A Review

**Published:** 2007-08-01

**Authors:** Hanno U Klemm, Olaf Franzen, Rodolfo Ventura, Stephan Willems

**Affiliations:** University Heart Center Hamburg, Hamburg, Germany

**Keywords:** Mapping, Electrophysiology, Myocardial Contraction, Cardiac Resynchronization Therapy

## Abstract

Heart failure as a result of a variety of cardiac diseases is an ever growing, challenging condition that demands profound insight in the electrical and mechanical state of the myocardium. Assessment of cardiac function has largely relied on evaluation of cardiac motion by multiple imaging techniques. In recent years electrical properties have gained attention as heart failure could be improved by biventricular resynchronization therapy. In contrast to early belief, QRS widening as a result of left bundle branch block could not be identified as a surrogate for asynchronous contraction.

The combined analysis of electrical and mechanical function is yet a largely experimental approach. Several mapping system are principally capable for this analysis, the most prominent being the NOGA-XP system. Electromechanical maps have concentrated on the local shortening of the reconstructed endocardial surface from end-diastole to end-systole. Temporal analysis of motion propagation, however, is a new aspect. The fundamental principles of percutaneous catheter based activation and motion assessment are reviewed. Related experimental setups are presented and their main findings discussed.

## Introduction

**Figure 1:**
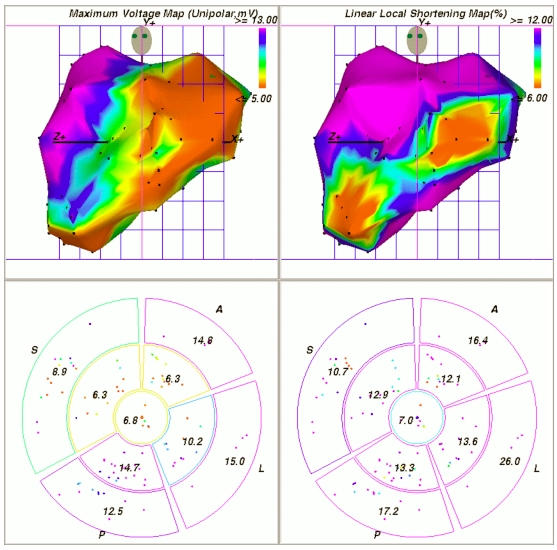
Left anterior oblique view of NOGA-XP left ventricular unipolar voltage (upper left) and linear local shortening (upper right) electromechanical maps. Data are obtained from a swine chronic infarction model after placement of an ameroid constrictor around the circumflex artery. Note that the apex points to the right. Colors represent peak-to-peak amplitudes of intracardiac electrocardiograms ranging from 5 mV (red) to 13 mV (purple), and linear local shortening ranging from 6% (red) to 12% (purple).The lower panels show 9-segment regional views comparable with sectors used by echocardiography to assess wall motion scores. The depicted numbers represent the average segmental values of unipolar voltage (lower left) and linear local shortening (lower right). A, anterior; S, septum; P, inferior-posterior; and L, lateral. Image courtesy of Dr. Korff Krause, Asklepios Clinic St. Georg, Hamburg, Germany.

The evaluation of cardiac motion has traditionally been based on cine-angiography and echocardiography. Both methods being essentially two-dimensional share inherent limitations, as approximations are needed to quantify basic parameters like stroke volume and ejection fraction. Since the mid-1990s a non-fluoroscopic navigation technique entered the catheterization suites around the world [1]. Ultra-low magnetic fields generated underneath and undisturbed by the patient's body can be detected by sensors embedded within deflectable catheters. The technique introduced as CARTO® was readily embraced by the cardiac electrophysiology community and is now utilized worldwide [2]. Shortly after its introduction, the capability of the system to reconstruct the inner boundaries of the left ventricle during systole and diastole was investigated. Under the name of NOGA® the first application of catheter based mapping of myocardial motion, so-called electromechanical mapping, became available [3]. It concentrated on the detection of myocardial scar formations by analyzing the change of the chamber geometry during contraction in combination with recorded intracardiac unipolar voltages [4-6](Figure 1). Only recently, analysis of motion propagation in combination with electrical activation was studied [7]. Activation-motion mapping analyzes the sequence of the electrical activation wave during sinus or paced rhythm and the resulting propagation of the motion wave. Areas of activation-motion mismatch defined either by passive motion, i.e. motion preceding electrical activation, or delayed contraction following early local depolarization can be identified.

The therapeutic options to treat advanced heart failure today include resynchronization therapy. Further insight into the mechanisms underlying asynchronous contraction due to different myocardial alterations can be expected from combined activation and motion analysis. Other experimental approaches like localized stem cell or growth factor application [8,9] may utilize the mapping technique for defining the initial state and improvements of cardiac function.

## Assessment of Myocardial Motion

**Figure 2:**
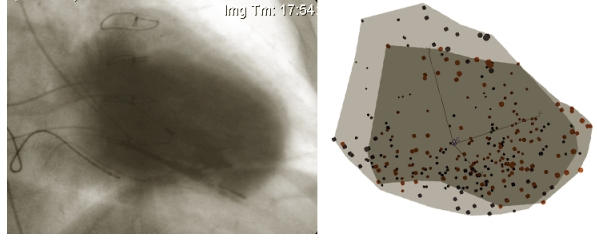
The left panel shows an overlay of two left ventricular angiography images taken at end diastole and end systole. The light contrasted area shows the end-diastolic volume, whereas the dark shaded area corresponds to end-systole. Apical akinesia and inferior hypokinesia due to remote myocardial infarction can be identified. It can also be noted that extended none-contracting areas are not substantially displaced by surrounding myocardium ("myocardial tethering") but remain stationary also in an absolute, fixed frame of reference, i.e. the fluoroscopy tube. The right panel displays points taken during a left ventricular activation motion map of the same patient. The light shaded area outlines point positions (blue) during end diastole. At end systole points have moved inward (red) and are outlined by the darker shaded area. The electromechanical map closely resembles the angiography images.

With the development of magnetic resonance tissue tagging an elegant method for a truly three-dimensional analysis and quantification of local myocardial function is now available [10]. From MRI studies we have learned that a complete description of myocardial motion requires three-dimensional strain analysis. Strain is a tool to quantify local motion independent of surrounding myocardium and is calculated for a small volume element by comparing the change in dimension during contraction to its original value in the relaxed state. Its three-dimensional principal components can be chosen to represent longitudinal shortening, myocardial radial thickening, and left ventricular torsion due to spiraling sub-epicardial and sub-endocardial fibers. In the normal heart, circumferential and longitudinal strains increase moderately from base to apex [12]. The heart, however, is fixed at the diaphragm, leading to an increase of absolute longitudinal motion from apex to base (Figure 2). The radial strain varies little from apex to base. A circumferential strain component remains elusive to detection by observing motion of the endocardium via catheter displacement.

### Motion trajectory in three-dimensional space

**Figure 3:**
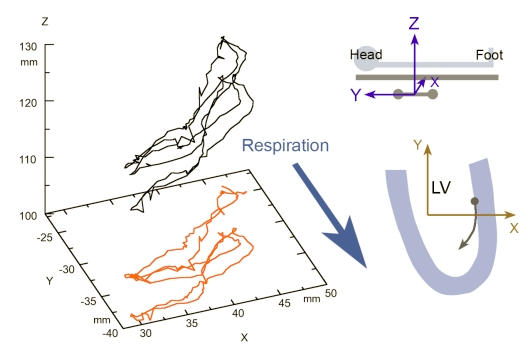
The left panel shows a three-dimensional plot of a catheter-tip trajectory during 3 consecutive beats. The red colored curve is a projection on the XY-plane to highlight respiratory effects. While the first two beats show little deviation respiration leads to a shift of the trajectory during the third beat predominantly along the y-axis. The frame of reference is fixed to the magnetic triangle as shown in the upper right panel. The sampled point displayed was taken from the left ventricular lateral free wall midway between base and apex of a healthy subject. The respiratory shift must be considered when sequentially sampled points are used for mechanical analysis.

For each point engaged by the mapping catheter the exact location in three-dimensional space is recorded. The series of locations of a single cardiac cycle makes up an elliptical shaped path with the major axis pointing towards the apex. With the recording of consecutive cycles, the trajectory takes up a helical shape. Each cycle gives rise to one loop of the helix (Figure 3). This phenomenon is a result of respiration, which causes a periodic motion of the entire heart. Respiration has been defined as a major source of inaccuracy of three-dimensional mapping, as it cannot be accounted for by ECG-gating [12,13]. For a single cycle, however, the effect is negligible. Furthermore, derivatives, e.g. velocity, are almost independent of respiratory effects. Problems are encountered when motion data are calculated that involve absolute positions of multiple, sequentially measured myocardial sites.

### Global vs. local myocardial motion: myocardial tethering

**Figure 4:**
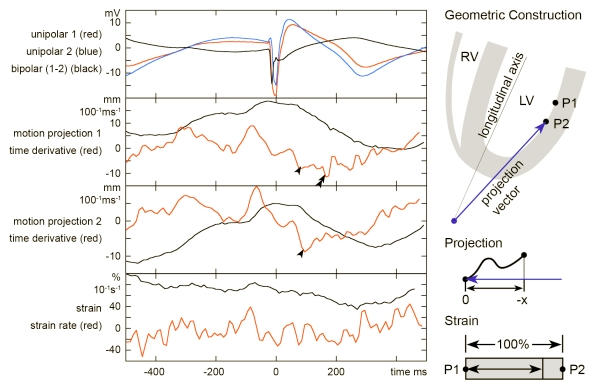
This compound figure shows a comparison between global and localized motion assessment. Catheter trajectories at two points at the left ventricular lateral free wall were recorded in a patient without structural heart disease. Points 1 and 2 were located along the longitudinal axis at 11mm separation as depicted in the geometric construction sketch. The left upper graph shows unipolar electrograms recorded from the tip electrode at these sites and a calculated bipolar electrogram. Projection of the three-dimensional motion on a vector yields motion curves shown for the two points. A 4-point time derivative was calculated. Arrowheads indicated points of minimum slope which can be used to define a local motion onset time. Please note the artificial minimum slope (double arrowheads) due to a small discontinuity of catheter motion at sample point 1. Therefore, manual revision of the motion onset time annotations is often necessary. Strain analysis as a local means of analyzing motion is shown in the lower graph. Lagrangian strain is defined as the change of dimension of a volume element in relation to its original size and usually given in percent. A longitudinal strain can be calculated from the distance of the two points during the cardiac cycle. The graph displays the separation as percentage of the initial distance of 11mm at end-diastole. A 4-point time derivative, i.e. strain rate, has also been calculated. Please not that strain is essentially a spatial derivative and the signal to noise ration is reduced. An additional temporal derivative yields data that can hardly be used to define motion onset. This seems to be a limitation of electromechanical mapping related to the accuracy of location and the sampling rate of three-dimensional catheter tracking.

The myocardium as a continuous structure transmits local deformation to neighboring areas. Any locally measured myocardial velocity therefore reflects active as well as passive motion. Obtaining strain data from closely spaced points by subtracting motion components from adjacent points can theoretically overcome this problem. Practically, spacing between points is random thereby introducing a varying error. Furthermore, spatial resolution of magnetic catheter tracking and beat-to-beat variability [14] is comparable to the amount of total displacement in hypokinetic areas. Although not systematically investigated, strain analysis by means of electro-anatomic mapping, requiring a spatial derivative, is probably not accurate when gradients in motion are low (Figure 4).

To overcome this problem, the NOGA-system employed a local shortening formula that averaged the change in distance between an index point and all its recorded surrounding points from late diastole to maximum systole [15]. To account for non-uniformity of sampled points a weighing function primarily emphasized points with a distance from 8 to 15mm.

The exact influence of tethering is unknown and is dependent on the elastic properties of the myocardium. Insights gained from angiography show that motion of hypo- or akinetic areas is dramatically reduced also in a static frame of reference. Therefore delineation of these areas is possible without strain analysis and correlates with bipolar voltage amplitude recorded at these sites [7]. A method used to quantify motion timing is derived from echocardiography using the observation that the endocardial surface moves from base to apex and radially towards the center. A vector is created from the apex towards the point under investigation. The three-dimensional trajectory of the catheter tip is projected onto the vector resulting in a one-dimensional motion component (Figure 4). This essentially reproduces the apical view obtained by trans-thoracic echocardiography for the 4- and 2-chamber standard planes. Velocities calculated as the first derivative of the motion component are similar to tissue Doppler measurements and can be evaluated accordingly. The temporal resolution of the magnetic mapping system is, however, less. While echocardiography samples tissue velocities at up to 300 frames per second, catheter trajectories are sampled at 100Hz. This introduces noise that increases when spatial or temporal derivatives are calculated.

### Catheter stability during the cardiac cycle

Stable contact of the catheter tip with a particular endocardial point during the cardiac cycle is of paramount importance when using the positional information for quantitative analysis of the myocardial contraction. In early studies Ben-Haim et al. [1] and Gepstein et al. [2,4] could demonstrate the stability of catheter position end end-diastole by using ECG-gating. Lessick et al. [14] conducted a detailed analysis of catheter stability during contraction. A catheter with a retractable needle was used to compare trajectories of the catheter tip placed conventionally with trajectories for which the tip was fixed to the myocardium by insertion of the needle. After correcting for respiration effects, a small average displacement of 1.33±0.61mm was found. Experimental data was obtained from healthy animals. It remains unknown, whether stability is conserved at scar areas showing a rather flat surface with fewer possibilities to hook the catheter tip.

For practical purposes certain approaches should be avoided for cardiac motion mapping. By bending the catheter by 180 degrees, i.e. using loops, the tip may loose contact with the endocardium during systole when the proximal shaft is shifted by contraction of the apex. The transseptal approach via the mitral valve is complicated by the mitral leaflets, papillary muscles, and tendons. A direct approach with little twisting of the catheter should be preferred.

### Stability criteria

Several criteria as published by Koronowski et al. [5] had to be met by sampled points to be eligible for hemodynamic analysis. Points were excluded if one of the following criteria were fulfilled: (1) a premature beat or a beat after a premature beat; (2) location stability, defined as a difference of > 5 mm in end-diastolic location of the catheter at 2 sequential heartbeats; (3) loop stability, defined as an average distance of > 5 mm between the location of the catheter at 2 consecutive beats at corresponding time intervals in the cardiac cycle; (4) cycle length that deviated > 10% from the median cycle length; (5) different morphologies of the local ECG at 2 consecutive beats; (6) local activation time differences of > 5 ms between 2 consecutive beats; and (7) different QRS morphologies of the body surface ECG.

For the analysis of motion timing some of the strict criteria can be modified. Reproducing the morphology of the intracardiac electrogram during consecutive beats is sufficient to ensure an unchanged position during diastole. Small beat-to-beat variations in the catheter tip trajectory are negligible since the absolute velocities remain almost unchanged.

### Defining the local motion onset time

As for the assessment of motion, timing ideally is calculated from local data. Strain rate being the time derivative of strain is used in conjunction with MRI [16]. Calculating strain, i.e. a spatial derivative, from catheter trajectories is limited (Figure 4). Calculating a time derivative from these data has an even lower signal-to-noise ratio. The method adopted for activation-motion mapping is based on established criteria for the evaluation of intra-ventricular asynchrony by tissue-Doppler [17,18]. Motion is indexed at the peak velocity which corresponds to the maximum down-slope of the projection motion curve. Although peak velocity is not necessarily consistent with motion onset, it is reproducible and the dispersion is a measure of asynchrony.

Other parameters derived from absolute displacement or strain analysis have been proposed. Zwanenburg et al. [16] used the onset of strain and fitted an interpolation function from which the onset time was approximated. The onset of velocity is less well defined but has been used in tissue Doppler studies [19].

## Determining myocardial activation

When compared with myocardial motion, defining an activation-time seems trivial. But we encounter analogous problems. In the experimental setting, intracellular recordings exactly define activation by the upstroke of the membrane potential. When compared with unipolar recordings obtained from the surface of homogeneous myocardial slabs a good approximation is the time at maximum down-slope of the unipolar electrogram [20,21]. Unipolar signals are susceptive to far-field signals, so-called electrotonic forces. Strictly speaking, unipolar signals are defined by recording far-fields [22]. Signals from nearby tissue can therefore obscure activation of low voltage areas. In analogy to myocardial tethering a localized analysis is needed. Bipolar signals have been widely used in electrophysiologic mapping to define local activation. In the ideal setting of homogenous tissue, the maximum down-slope definition of unipolar signals corresponds to the peak of the bipolar signal. In the diseased heart, broad and fractionated potentials are often encountered [23]. In these potentials the maximum amplitude can be located at multiple points within the signal. Also slope measurements may not give consistent results. Therefore, the annotation of the local activation time to the onset of the bipolar electrogram is a convenient method that has gained widespread use in electrophysiology and was also adapted for activation motion analysis [7].

## Related experimental setups

Early studies of combined activation and motion analysis concentrated on a limited area of the left ventricle. Prinzen et al. [24] measured the dispersion between electrical and mechanical activation at the anterior wall in open chest dogs. They found a progressive increase in the interval between electrical activation and onset of fiber shortening the later an area was depolarized. The potentiality of determining the temporal contractile response initiated by a specific excitation wave has led to the development of computational models by Usyk and McCulloch [25,26]. Interestingly, although a constant time delay of 8ms was set from electrical activation to the development of fiber tension; large variations in the delay from fiber tension to systolic shortening were detected. Also, shortening was noted preceding fiber tension and depolarization by as much as 50ms. These computational findings relate to the clinical observation that electrical asynchrony is not necessarily predictive for mechanical asynchrony. In an experimental setup studying the canine heart, the group of McVeigh et al. [27,28] combined the technique of epicardial unipolar mapping with MRI tissue tagging. Electrograms were retrieved from an implanted sock-electrode [29] that covered the epicardium with an evenly spaced array of 247 electrodes. Gadolinium markers were used to identify the electrodes on the MRI images. This variant of electromechanical mapping was also studied for the canine post-myocardial infarction heart [30]. In addition to motion analysis, myocardial scarring could be identified using delayed hyper-enhancement MRI. Activation was delayed in the infarct border zone. The region surrounding the border showed normal electrical activation. Strain maps, however, revealed abnormal stretch and loss of normal strain gradients extending far beyond the infarct area. Mechanical coupling of healthy and infarcted myocardium seems to impose a crucial stress on the remaining contractile fibers. The varying dependence of mechanical failure regarding electrical activation might be fundamental to the understanding of heart failure refractory to resynchronization therapy [31]. Due to the need to implant an epicardial sock-electrode requiring thoracotomy, this approach largely remains experimental.

A replacement of epicardial contact mapping could be the use of magnetocardiography sensor arrays. Using this approach electrical potentials on the epicardial surface are calculated from signals recorded on the patient's skin. This method was adopted by Jeremic et al. [32] and prompts for further investigation in this elegant and non-invasive technique.

## Currently available systems for activation and motion mapping

Today, the most dedicated platform to assess left ventricular mechanics is the NOGA-XP [1,2] system now distributed by Biologics Delivery Systems, Diamond Bar, CA. Although analysis and map-display of motion timing have not been implemented these data are readily available. Export of trajectory data is possible and the time-course of local shortening can be displayed. In addition several criteria to obtain stable catheter positions are available online. Biosense Webster's CARTO, although build on the same technology, does not offer the export of motion data. These remain hidden in the DICOM backup-files.

The Ensite Array and NavX technologies (St. Jude Medical former Endocardial Solutions Inc., St. Paul, MN) [33,34] that use high frequency electric fields to locate any type of EP catheter have the potential to be used for motion analysis in the future. As with NOGA-XP, a wide spectrum of data including motion trajectories can be exported. The sampling rate is a complex function from a baseline rate of 1200Hz as multiple samples are combined to result in a complete set of coordinates. An effective rate of 93Hz is feasible. A limitation  of the current software is that only the current location in space and no trajectory is saved with each point acquired in a Landmarks map. Generation of a motion map would therefore require the individual recording of Enguide-motion for each point and subsequent export. A further problem is the in-homogeneity of the generated electric fields that has to be corrected [12]. Compensation algorithms are expected for the near future. No means for analyzing motion are available by the software as of version 6.

The Realtime Position Management system (Boston Scientific former Cardiac Pathways, Sunnyvale, CA) [35,36] utilizes ultrasound which, due to the theoretical high sampling rate, has good potentiality for motion analysis. The system, however, allows for 15 frames / second in realtime mode, which is not sufficient for mechanical analysis.

## Where to go from here?

Mismatches between cardiac activation and motion propagation are of growing interest in the evaluation of heart failure patients who are candidates for resynchronization therapy. In addition, the role of this mismatch in the evolution of heart failure is largely unknown.

Electromechanical remodeling in the ischemic heart has probably fundamental differences to the process occurring in idiopathic dilated cardiomyopathy. A change of activation and motion propagation may as well serve as a sensitive surrogate for the state of the disease and consequently as a marker for reverse remodeling. The latter is of importance in the delivery of stem cells or growth factors which is feasible using NOGA [8,9,37,38]. For patients receiving invasive follow-up monitoring of changes in contraction pattern may be more sensitive than global estimation of pump function by virtue of the ejection fraction.

## Conclusion

Activation and motion mapping is a valuable extension of currently available mapping techniques. Several catheter tracking systems meet the technical prerequisites to establish motion analysis. Most of the necessary online tools to sample catheter motion have already been included in the NOGA-XP system. Studies are warranted to define the role of activation and motion propagation in the definition of heart failure and cardiac remodeling. Being largely experimental today, the step from bench to bedside seems possible in the near future.
